# The Predictive Value of Infant-Specific Preoperative Pulmonary Function Tests in Postoperative Pulmonary Complications in Infants with Congenital Heart Diseases

**DOI:** 10.1155/2019/2781234

**Published:** 2019-03-03

**Authors:** Xin Liu, Feng Qi, Jichang Chen, Songrong Yi, Yanling Liao, Zhuoxin Liang, Jing Zhou, Yan Feng

**Affiliations:** ^1^Department of Pediatrics, Liuzhou Maternity and Child Health Hospital, Liuzhou 545001, China; ^2^Department of Cardiac Surgery, the 2nd Affiliated Hospital of Harbin Medical University, Harbin 150086, China

## Abstract

**Background and Objective:**

To investigate the relationship between infant-specific preoperative pulmonary function tests (PFTs) and postoperative pulmonary complications (PPCs) in infants with congenital heart diseases (CHDs).

**Methods:**

Patients of 1-3 years of age who received surgical treatment for CHDs from January 1^st^, 2009, to December 31^st^, 2017, were retrieved. Records of preoperative PFTs, methods of operation, anesthesia procedures, intraoperative vital signs, respiratory support modalities, and PPCs was retrieved and analyzed.

**Results:**

122 infants met the preset inclusion criteria, including 72 males and 50 females. There were 76 cases of thoracotomy and 46 cases of cardiac catheterization. The overall incidence of PPCs was 15.6%, including 19.7% after thoracotomy and 8.7% after cardiac catheterization, respectively (*p* > 0.05). The incidence of PPCs was 35.4% or 2.7% in infants with a rapid or a normal respiratory rate, respectively; 42.1% or 3.6% in infants with an abnormal or a normal time to reach peak tidal expiratory flow versus the total expiratory time (TPTEF/TE), respectively; 39.0% or 3.7% in infants with an abnormal or a normal volume to peak expiratory flow versus the total expiratory volume (VPEF/VE), respectively; and 46.9% or 4.4% in infants with a decreased or a normal lung compliance, respectively (*p* < 0.01 in all comparisons).

**Conclusions:**

The preoperative abnormal changes in respiratory rate, TPTEF/TE, VPEF/VE, and lung compliance are indicative of the risk of PPCs.

## 1. Introduction

Congenital heart diseases (CHDs) refer to a group of malformations due to abnormal cardiovascular development in fetal period and account for nearly 1/3 of all major congenital anomalies [1]. About 1.35 million newborns were born with CHDs worldwide every year, with the highest incidence rate being 9.3 per 1,000 live births reported in Asia. CHDs are the leading causes of birth defect-associated infant illness and death. About 25% of patients with CHDs need surgical or interventional therapies during neonatal period or infancy [2]. However, postoperative pulmonary complications (PPCs), such as respiratory infection or respiratory failure, remain common after surgical treatments [3], leading to prolonged hospital stay and even death.

At present, it is believed that adults with compromised lung functions are predisposed to PPCs [4]. Therefore, preoperative pulmonary function tests (PFTs) have been employed as important indices to evaluate the prognosis of PPCs in adults. However, the correlation between preoperative PFTs and PPCs in CHDs infants remains unclear. On the one hand, the PFTs performed in infants and adults are quite different, due to practical issues [5]. On the other hand, there are few studies analyzing preoperative PFTs in infants. In the present study, we analyzed infant-specific preoperative PFTs in CHDs infants treated with surgeries and their relationship to PPCs.

## 2. Methods

This retrospective cohort study was performed at Liuzhou Maternal and Child Health Hospital, a tertiary hospital and the regional specialized center for the treatment of CHDs in south China. Institutional review board approval was obtained before the start of the study. Included patients should meet all of the following criteria: (1) had complete admission records, postoperative course records, and pulmonary function tests and were admitted between January 1, 2009, and December 31, 2017, for surgical treatments of CHDs; (2) met the diagnostic criteria of CHDs [6] and indications of surgical treatment [7]; (3) be 1-3 years old when admitted; and (4) had no chromosomal abnormalities, or other chronic diseases, such as diabetes or endocrine disorders. Patient information about preoperative routine examination, preoperative PFTs, anesthesia procedures, intraoperative vital signs, respiratory support modalities, and PPCs was retrieved and analyzed.

The surgeries were performed by the same group of surgeons, with experience of similar surgeries for over 5 years before the start date of this study. The diagnosis of PPCs included respiratory infections (bronchiolitis and pneumonia), respiratory failure, atelectasis, pneumothorax, hypoxemia, bronchospasm, or postoperative respiratory support in ICU for more than 2 weeks after operation [8].

The preoperative PFTs were measured as described previously [9], with a few modifications. Briefly, all patients received oral choral hydrate (0.3-0.5 ml/kg) to be kept asleep during PFTs, which were performed at least 4 hours after feeding to avoid abdominal distension or vomiting. Temperature and humidity in the test room were maintained at 22°C and 40%, respectively. Infants lay flat on the test bed on their back, with their mouth and nose covered with an airtight mask. The PFTs were measured by a trained physician after smooth breath had been established, using a MasterScreen BabyBody plethysmograph (Jaeger, Germany), and 15-20 cycles of tidal breathing were recorded, with 5 repeats. The mean value of the 5 PFTs was calculated and used for analyses. An increased preoperative respiratory rate was defined as above 40 times/min [10]. The time to reach peak tidal expiratory flow versus the total expiratory time (TPTEF/TE) and the volume-to-peak expiratory flow versus the total expiratory volume (VPEF/VE) less than 30%, or above 50% were defined as abnormal, respectively [11, 12]. A lung compliance less than 10 ml/kPa/kg was defined as decreased [13]. Inspiratory to expiratory thoracoabdominal (TA) displacement ratio (TIF50/TEF50, where TIF50 is tidal inspiratory TA displacement rate at 50% of inspiratory displacement and TEF50 is tidal expiratory TA displacement rate at 50% of expiratory displacement), peak expiratory flow (PEF), and the time to peak tidal expiratory flow (TPTEF) were also measured.

### 2.1. Statistical Analysis

Discrete data were expressed as number of cases (percentages) and analyzed using the *χ*^2^ test or Fisher's exact test, along with odds ratio (OR) and 95% confidence interval (95% CI), whichever was applicable. Continuous data were shown as mean ± standard deviation (SD) and were analyzed using the *t* test. The area under the receiver operating characteristic (ROC) curve was used to show the value of prediction. SPSS 24.0 (IBM Corp, Armonk, NY) was used for statistical analysis. A two-tailed *p* < 0.05 is considered significantly different.

## 3. Results

A total of 122 cases were retrieved according to the inclusion criteria, including 72 males and 50 females. There were 76 cases of thoracotomy and 46 cases of cardiac catheterization. There was no significant difference in age, gender, height, or weight between the two surgical groups (*p* > 0.05 in all comparisons), except in the duration of operation (*p* < 0.01, Table 1).

### 3.1. Incidence of PPCs in CHDs of Different Surgical Groups

There were 32 cases of patent ductus arteriosus (PDA, 3 PPC cases in 28 cases of the catheterization group and 1 PPC case in 4 cases of the thoracotomy group, *p* > 0.05), 4 cases of atrial septal defect (ASD, 0 PPC case in 1 case of the catheterization group and 0 PPC case in 3 cases of the thoracotomy group, *p* > 0.05), 55 cases of ventricular septal defect (VSD, 1 PPC cases in 14 cases of the catheterization group and 8 PPC cases in 41 cases of the thoracotomy group, *p* > 0.05), 6 cases of pulmonary stenosis (PS, no case of the catheterization group and 0 PPC case in 6 cases of the thoracotomy group, *p* > 0.05), 5 cases of tetralogy of Fallot (TOF, no case of the catheterization group and 1 PPC case in 5 cases of the thoracotomy group, *p* > 0.05), and 20 cases of ASD + VSD (0 PPC case in 3 cases of the catheterization group and 5 PPC cases in 17 cases of the thoracotomy group, *p* > 0.05). The overall incidence of PPCs was 15.6%, with 8.7% after cardiac catheterization and 19.7% after thoracotomy, respectively, without a significant difference (*p* > 0.05, Table 2).

### 3.2. Relationship between Preoperative PFTs and PPCs

The incidence of PPCs was 29.3% or 3.1% in infants with a rapid or a normal respiratory rate, respectively (positive predictive value, or PPV = 89.5%, and negative predictive value, or NPV = 60.2%, *p* < 0.01); 33.3% or 4.1% in infants with an abnormal or a normal TPTEF/TE, respectively (PPV = 84.2% and NPV = 68.9%, *p* < 0.01); 31.4% or 4.2% in infants with an abnormal or a normal VPEF/VE, respectively (PPV = 84.2% and NPV = 66.0%, *p* < 0.01); and 35.7% or 5% in infants with a decreased or a normal lung compliance, respectively (PPV = 79.0% and NPV = 73.8%, *p* < 0.01, Table 3). For PFTs without clear normal ranges, such as TIF50/TEF50, PEF, or TPTEF, there were no significant differences between the PPC group and the non-PPC group (*p* > 0.05, Table 4).

**Figure 1:**
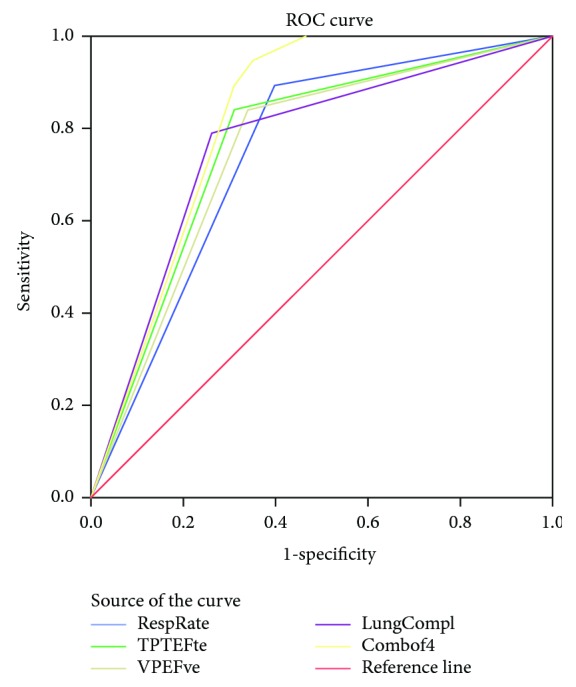
Receiver operating characteristic (ROC) curve of respiratory rate (blue), TPTEF/TE (green), VPEF/VE (pale), lung compliance (purple), and combination of respiratory rate, TPTEF/TE, VPEF/VE, and lung compliance patients (yellow).

Using ROC curve analysis, we found that the area under the curve of respiratory rate, TPTEF/TE, VPEF/VE, and lung compliance were 0.748 ± 0.054 (95% CI: 0.643, 0.854, *p* < 0.01), 0.766 ± 0.056 (95% CI: 0.655, 0.876, *p* < 0.001), 0.751 ± 0.057 (95% CI: 0.639, 0.863, *p* < 0.01), and 0.764 ± 0.060 (95% CI: 0.646, 0.881, *p* < 0.001), respectively, whereas combination of the 4 positive PFTs (Combof4) improved the predictive value to 0.821 ± 0.038 (95% CI: 0.746, 0.896, *p* < 0.001, Figure 1 and Table 5). Furthermore, we found that combination of the 4 positive PFTs can be included in a logistic regression equation for prediction of PPCs, i.e., *p* = 1/[1 + e^−(−4.763 + 0.887Combof4)^],

## 4. Discussion

The pathogenesis of PPCs in infants has not been clearly characterized yet. Available studies in adults show that PPCs originate differently from respiratory infections without surgeries [14]. Atelectasis and respiratory infections seem to be related to disruption of the normal activity of the respiratory muscles during anesthesia procedures. Chest or abdomen surgeries in adults can cause diaphragmatic dysfunction as well as reduction of vital capacity, tidal volume, or forced expiratory volume in one second (FEV1), resulting in atelectasis. Furthermore, diaphragmatic dysfunction, postoperative pain, anesthetics, and postsurgical stress all suppress the clearance of secretions in the respiratory tract, leading to atelectasis or respiratory infections [15].

The preoperative PFTs of adult patients are among the key indices that influence the short-term prognosis after the surgery [16]. However, preoperative PFTs have not been well applied in the field of pediatrics. It has been shown that many of the lung function parameters, such as total lung capacity (TLC), residual volume (RV), functional residual capacity (FRC), forced vital capacity (FVC), and forced expiratory flows at 25, 50, 75, 85, and between 25% and 75% of expired FVC (FEF_25_, FEF_50_, FEF_75_, FEF_85_, and FEF_25–75_, respectively) are all positively related to infant length, whereas RV/TLC, FRC/TLC, and FEF_25–75_/FVC are all negatively related to infant length [17]. Therefore, we employed TPTEF/TE and VPEF/VE, which are more infant-specific [11]. In order to minimize the difficulty to carry out the measurements in infants of this age group, as well as to obtain results of good quality and reproducibility, oral choral hydrate (0.3-0.5 ml/kg) was given to all participants to keep them asleep during PFTs, according to previous studies [9].

Unexpectedly, in the present study, although the duration of operation is significantly longer in thoracotomy, we did not find a significant difference in the incidence of PPCs between the thoracotomy and the catheterization patients. This coincides with a previous report showing that the length of surgery is only a risk factor for PPCs when it is more than 3 hours [16]. Therefore, we pooled the patients from the two surgical groups and increased our stratified sample size. We found that the incidence of PPCs was significantly higher in infants with an abnormal respiratory rate, or with an abnormal VPEF/VE, or with an abnormal TPTEF/TE, or with a decreased lung compliance (all *p* < 0.01). The positive and negative predictive values are good for all of the 4 indices (Table 3), with an even better predictive value when these 4 PFTs are considered altogether, showing the reliability of infant-specific preoperative PFTs in the prediction of PPCs in infants. VPEF/VE and TPTEF/TE have been shown to be significantly lower in asthmatic children and significantly increased after salbutamol inhalation [12], thus their predictive value in the development of PPC might be rooted in the functional reserve of the respiratory tract.

Although it has been reported in elder children (>7 years old) that TIF50/TEF50 was significantly higher in asthma cases [18], and PEF variation was positively associated with asthma symptoms [19], due to the difference in ages and methods of measurement, we did not find any difference in TIF50/TEF50 or PEF between the PPC group and the non-PPC group. Also coinciding with previous reports analyzing TPTEF in airway obstruction in infants [20], we did not find a significant difference in TPTEF between the PPC group and the non-PPC group.

After the assessment of preoperative PFTs, special attention should be paid to infants at high risks during preoperative preparation to improve respiratory functions, including pulmonary ventilation reserves and compliance of lung to prepare for the incoming surgery. Surgeries are recommended only after the lung function indices have significantly improved. Lung function should also be protected during and after surgeries, such as reducing the time of surgery and facilitating the drainage of airway secretions [21]. Our retrospective study design and relatively small size of sample are limitations of our present study, and prospective studies involving more participants are needed in the future.

In summary, infant-specific preoperative PFTs are key prognostic predictive factors for CHD corrective surgeries. Patients with abnormal respiratory rate, VPEF/VE, TPTEF/TE, or lung compliance are at high risk for the development of PPCs. Those infant-specific PFTs have potential values in the decision of the mode and range of surgery, as well as the mode and depth of the anesthesia procedures, in order to reduce PPCs and postoperative mortality.

## Figures and Tables

**Table 1:** Clinical characteristics of enrolled patients.

Cases	Catheterization	Thoracotomy	OR (95% CI)^#^	*p* value
Male^∗^	25	47	0.73 (0.35, 1.54)	0.67
Duration of surgery (hr)^∗∗^	0.48 ± 0.03	2.25 ± 0.21	—	<0.01
Height (cm) ^∗∗^	76.23 ± 4.15	77.51 ± 8.23	—	0.33
Weight (kg) ^∗∗^	9.93 ± 1.32	9.78 ± 1.73	—	0.61
Age (months) ^∗∗^	18.12 ± 4.65	18.22 ± 9.18	—	0.95
Total	46	76	—	—

^∗^ *χ* ^2^ test, number of patients. ^∗∗^*t* test, mean ± SD. ^#^Odds ratio (95% confidence interval).

**Table 2:** Cases of congenital heart diseases in different surgical groups.

Cases	Catheterization	Thoracotomy	OR (95% CI)	*p* value
PDA	28 (3)	4 (1)	2.78 (0.21, 35.95)	0.43
ASD	1 (0)	3 (0)	—	1
VSD	14 (1)	41 (8)	3.15 (0.36, 27.76)	0.42
PS	0 (0)	6 (0)	—	1
TOF	0 (0)	5 (1)	—	1
ASD + VSD	3 (0)	17 (5)	—	0.54
Total	46 (4)	76 (15)	2.58 (0.8, 8.33)	0.13

Fisher's exact test for all comparisons. Numbers in brackets represent PPC cases. PDA: patent ductus arteriosus; ASD: atrial septal defect; VSD: ventricular septal defect; PS: pulmonary stenosis; TOF: tetralogy of Fallot.

**Table 3:** Relationship between preoperative pulmonary function tests (with normal range) and postoperative pulmonary complications.

Groups	PPC	Non-PPC	OR (95% CI)	*p* value	PPV	NPV
Respiratory rate	19 (17)	103 (41)	12.9 (2.8, 58.6)	<0.01	89.5%	60.2%
TPTEF/TE	19 (16)	103 (32)	11.8 (3.2, 43.5)	<0.01	84.2%	68.9%
VPEF/VE	19 (16)	103 (35)	10.4 (2.8, 38.0)	<0.01	84.2%	66.0%
Lung compliance	19 (15)	103 (27)	10.6 (3.2, 34.6)	<0.01	79.0%	73.8%

Fisher's exact test for all comparisons. Numbers in brackets represent abnormal cases. PPC: preoperative pulmonary function; PPV: positive predictive value; NPV: negative predictive value.

**Table 4:** Relationship between preoperative pulmonary function tests (without normal range) and postoperative pulmonary complications.

Groups	PPC (19)	Non-PPC (103)	*p* value
TIF50/TEF50	80.3 ± 15.6	86.5 ± 37.4	>0.05
PEF (ml/s)	99.1 ± 31.4	108.5 ± 14.9	>0.05
TPTEF (s)	0.27 ± 0.21	0.32 ± 0.21	>0.05

Numbers in brackets represent cases in PPC group or no-PPC group.

**Table 5:** Details of area under the ROC curve in Figure 1.

Area under the curve					
Test result variable(s)	Area	Std. error^a^	Asymptotic sig.^b^	Asymptotic 95% confidence interval	
				Lower bound	Upper bound
RespRate	.748	.054	.001	.643	.854
TPTEFte	.766	.056	.000	.655	.876
VPEFve	.751	.057	.001	.639	.863
LungCompl	.764	.060	.000	.646	.881
Combof4	.821	.038	.000	.746	.896

## Data Availability

Original data could be obtained by contacting the corresponding author.

## References

[1] van der Linde D., Konings E. E. M., Slager M. A. (2011). Birth prevalence of congenital heart disease worldwide: a systematic review and meta-analysis. *Journal of the American College of Cardiology*.

[2] Centers for Disease Control and Prevention (2018). Congenital Heart Defects (CHDs). https://www.cdc.gov/ncbddd/heartdefects/data.html.

[3] Bandla H. P. R., Hopkins R. L., Beckerman R. C., Gozal D. (1999). Pulmonary risk factors compromising postoperative recovery after surgical repair for congenital heart disease. *Chest*.

[4] Fisher B. W., Majumdar S. R., McAlister F. A. (2002). Predicting pulmonary complications after nonthoracic surgery: a systematic review of blinded studies. *The American Journal of Medicine*.

[5] Seed L., Wilson D., Coates A. L. (2012). Children should not be treated like little adults in the PFT lab. *Respiratory Care*.

[6] Ferencz C., Rubin J. D., Mccarter R. J. (1985). Congenital heart disease: prevalence at livebirth. The Baltimore-Washington Infant Study. *American Journal of Epidemiology*.

[7] Moss A. J., Adams F. H., Maloney J. V., Longmire W. P., O'Loughlin B. J. (1958). Congenital cardiac defects-indications for surgical repair. *California Medicine*.

[8] Jammer I., Wickboldt N., Sander M. (2015). Standards for definitions and use of outcome measures for clinical effectiveness research in perioperative medicine: European Perioperative Clinical Outcome (EPCO) definitions: a statement from the ESA-ESICM joint taskforce on perioperative outcome measures. *European Journal of Anaesthesiology*.

[9] Lesnick B. L., Davis S. D. (2011). Infant pulmonary function testing: overview of technology and practical considerations--new current procedural terminology codes effective 2010. *Chest*.

[10] Normal Values in Children (2018). https://www.aclsmedicaltraining.com/normal-values-in-children/.

[11] Stocks J., Dezateux C. A., Jackson E. A., Hoo A. F., Costeloe K. L., Wade A. M. (1994). Analysis of tidal breathing parameters in infancy: how variable is TPTEF:TE?. *American Journal of Respiratory and Critical Care Medicine*.

[12] Carlsen K. H., Lødrup Carlsen K. C. (1994). Tidal breathing analysis and response to salbutamol in awake young children with and without asthma. *European Respiratory Journal*.

[13] Gerhardt T., Hehre D., Feller R., Reifenberg L., Bancalari E. (1987). Pulmonary mechanics in normal infants and young children during first 5 years of life. *Pediatric Pulmonology*.

[14] Warner D. O. (2000). Preventing postoperative pulmonary complications: the role of the anesthesiologist. *Anesthesiology*.

[15] Wilcox S., Gaissert H. (2016). Diaphragmatic dysfunction after thoracic operations. *The Journal of Thoracic and Cardiovascular Surgery*.

[16] Smetana G. W. (1999). Preoperative pulmonary evaluation. *The New England Journal of Medicine*.

[17] Castile R., Filbrun D., Flucke R., Franklin W., McCoy K. (2000). Adult-type pulmonary function tests in infants without respiratory disease. *Pediatric Pulmonology*.

[18] Hmeidi H., Motamedi-Fakhr S., Chadwick E. (2017). Tidal breathing parameters measured using structured light plethysmography in healthy children and those with asthma before and after bronchodilator. *Physiological Reports*.

[19] Brand P. L. P., Duiverman E. J., Waalkens H. J., van Essen-Zandvliet E. E. M., Kerrebijn K. F., the Dutch CNSLD Study Group (1999). Peak flow variation in childhood asthma: correlation with symptoms, airways obstruction, and hyperresponsiveness during long-term treatment with inhaled corticosteroids. Dutch CNSLD Study Group. *Thorax*.

[20] Hevroni A., Goldman A., Blank-Brachfeld M., Abu Ahmad W., Ben-Dov L., Springer C. (2018). Use of tidal breathing curves for evaluating expiratory airway obstruction in infants. *Journal of Asthma*.

[21] Apostolakis E. E., Koletsis E. N., Baikoussis N. G., Siminelakis S. N., Papadopoulos G. S. (2010). Strategies to prevent intraoperative lung injury during cardiopulmonary bypass. *Journal of Cardiothoracic Surgery*.

